# Therapeutic Role and Ligands of Medium- to Long-Chain Fatty Acid Receptors

**DOI:** 10.3389/fendo.2014.00083

**Published:** 2014-06-02

**Authors:** Takafumi Hara, Atsuhiko Ichimura, Akira Hirasawa

**Affiliations:** ^1^Department of Pharmacogenomics, Graduate School of Pharmaceutical Sciences, Kyoto University, Kyoto, Japan; ^2^Department of Molecular Medicine and Therapy, Tohoku University Graduate School of Medicine, Sendai, Japan

**Keywords:** FFA1, FFA4, medium- to long-chain fatty acid, energy metabolism

## Abstract

Medium- and long-chain free fatty acids (FFAs) are energy source for whole body and biological metabolites and components. In these decades, some research groups have reported that the biological functions of medium- to long-chain FFAs are exerted through G-protein coupled receptor designated free fatty acid receptor (FFAR). As the medium- to long-chain FFAs-activated FFARs, FFA1 and FFA4 are reported to be expressed widely in whole body and regulate various physiological processes. FFA1 expressed in pancreatic β-cells has been shown to be involved in insulin secretion. FFA4 expressed in intestine, adipocytes, and macrophages has been shown to be involved in incretin secretion, differentiation, and anti-inflammatory effect, respectively. These physiological functions have been focused on the treatment of metabolic disorders. In addition, these receptors have been also reported to be expressed in several other tissues such as intestine for FFA1, and tongue and stomach for FFA4. The recent functional studies indicated that they also contributed to energy homeostasis. Further, the number of synthetic compounds of FFA1 and FFA4 strongly promoted the physiological characterization of the receptors and their own therapeutic utility. In this article, we will discuss the recent progress regarding the therapeutic potential of these receptors and its ligands.

## Introduction

Free fatty acids (FFAs) are known to act as the critical energy source in the whole body. In addition, FFAs also act as signal molecules in various physiological reactions (1, 2). As one of these mechanisms, G-protein-coupled receptors (GPCRs) activated by FFAs designated as free fatty acid receptor (FFAR) family have played important role especially in energy metabolism (3). Among FFAR family, FFA1 (known as GPR40) and FFA4 (known as GPR120) are classified as medium- to long-chain fatty acid-activated receptors (4, 5). On the other hand, FFA2 and FFA3 are classified as short-chain fatty acid-activated receptors (refer to each topic) (6, 7). In terms of natural ligands for FFA1 and FFA4, medium- and long-chain fatty acids that showed various physiological functions generally and contained 6–12 and more than 12 carbon chains are mainly provided by food digestion and lipolysis in adipose tissues. As unsaturated fatty acids that include double bond are not supplied in biosynthesis in human, we have to take in these unsaturated fatty acid as food intake. A number of studies for FFA1 and FFA4 revealed that these two receptors have been contributed to regulate energy metabolism and metabolic diseases such as type 2 diabetes and obesity. In this review, we summarize the therapeutic utility of medium- and long-chain fatty acid receptors, FFA1 and FFA4.

## Therapeutic Potential of FFA1 and FFA4 Physiological Functions Related to Energy Metabolism

Among FFARs, FFA1 and FFA4 are classified as medium- to long-chain FFARs. The ligand property of these two receptors was similar to each other; however, the expression profile is different in several tissues (4, 5). The physiological functions of FFA1 and FFA4 related to the energy metabolism regulation are as follows.

### FFA1

Free fatty acid 1 expressed in pancreatic β-cell and intestine has been considered to have therapeutic utility. FFA-induced glucose-stimulated insulin secretion (GSIS) in β-cells via FFA1 has been reported by Itoh et al. (4). Using FFA1 KO and TG mice, FFA1 exhibited protective effect against chronic and/or excess stimulation of glucose-induced toxic effect on β-cells (8–10). In addition, depolymerization of F-actin and the activation of protein kinase D1 (PKD1) are contributed to the FFA1-mediated insulin secretion from β-cells (11). Further, Flodgren et al. reported that not only in β-cells but also α-cells also expressed FFA1 and contributed to glucagon secretion (12). In intestine, FFA1 expression was also confirmed in the gastric inhibitory polypeptide (GIP) and glucagon like peptide-1 (GLP-1) incretin hormones secreting cells such as the intestinal K and L cells (13, 14). In addition, intestinal I cells that express cholecystokinin (CCK) also expressed FFA1 were reported by Liou et al. (15). Therefore, FFA1 might regulate insulin secretion not only in direct but also in indirect mechanism.

### FFA4

Free fatty acid 4 expressed in intestine, adipose tissue, and taste buds is considered as therapeutic target for metabolic disorders. Similar to FFA1 expression in intestine, FFA4 is expressed in intestinal L-cell that can secrete GLP-1 (5). In adipose tissue, FFA4 has been contributed to regulate adipose differentiation and GLUT-4 translocation that regulates glucose incorporation (16, 17). The effect of FFA4 on energy metabolism was showed in GPR120 KO mice study (18). Further, FFA4 expressed in macrophages was reported to regulate inflammatory responses (17). On the other hand, in taste buds, FFA4 expression was confirmed in type 1 and 2 taste bud cells (19, 20). Since the FFA stimulation of these cells activated taste nerve response, fat preferences might be regulated via FFA4. These findings provided us the possibility for the therapeutic utility of FFA1 and FFA4.

## Synthetic Ligands and Its Therapeutic Utility

### FFA1

To regulate these physiological functions, more selective ligands are expected to be developed for therapeutic candidates. For FFA1, a number of selective agonists have been reported. The expected pharmacological property of the compound is the selectivity compared to other FFARs, the potency compared to natural ligands, and efficacy not only in *in vitro* but also in *in vivo* experiments. NCG75, GW9508, and TAK-875 have been reported as selective and potent agonists. However, these compounds showed similar effect on insulin secretion compared to the endogenous FFA ligands in insulin-secreting cell line such as INS-1 and MIN6 cells (21–23). Recently, TAK-875 has been reported to be failed in clinical trial owing to the side effect such as liver toxicity. Therefore, unknown signaling mechanisms might contribute to regulate the physiological functions. To obtain the beneficial effect of FFA1, we need to understand more precise mechanisms in cell line levels.

### FFA4

For FFA4, we identified a partial agonist among the natural compounds derived from fruiting bodies of *Albatrellus ovinus* (24). Further, we also identified a series of synthesized compounds based on PPARγ-agonist thiazolidinediones. Among these compounds, a selective agonist NCG21 showed selectivity for FFA4 compared to FFA1 (25, 26). TUG-891 developed by Hudson et al. showed the most potent agonistic activity that activated not only in G-protein but also in β-arrestin-dependent pathways (27, 28). Metabolex was developed as selective agonist for FFA4 and the pharmacological properties were examined not only in cultured cells but also in animal models (29).

In addition, isoindolin-1-one series and phenyl-isoxazol-3-ol series developed by Banyu Pharmaceutical Co. Ltd. showed potent selectivity for FFA4 compared to FFA1 with nanomolar order potency (30, 31).

## Further Challenge of the FFA1 and FFA4 Research for Therapeutic Application

As described in previous section, the physiological and pharmacological functions of these two receptors have increasingly revealed. The synthetic ligands that showed the usefulness not only for *in vitro* but also for *in vivo* study have been also increased. However, to proceed these findings to develop the pharmaceutical agents, we should answer the several questions about the molecular mechanisms of these receptors. The signal transduction mechanisms of these two receptors have been reported that FFA1 coupled to G_q_ protein but not the G_i/o_ or G_s_, and FFA4 coupled to G_q_ family and β-arrestin pathways (4, 5, 17, 32). However, the precise mechanism that connects a signal pathway and a specific physiological function has not been well-understood yet. The dimerization of receptors has been reported to regulate the affinity of the ligand, signaling pathway, and related physiological functions (33). Since FFARs including short-chain fatty acid receptors are expressed in the same tissues such as pancreatic β-cells, intestine and immune cells, the dimerization of these receptors might be formed in cell surface, and the physiological functions of each receptor might be regulated. On the other hand, the protein expression profile should be examined more precisely because several studies reported that the receptor expression is controversial. Although the receptor expression in the tissues is evaluated by mRNA levels in some studies, mRNA levels do not always reflect the protein level. Various commercial antibodies for FFA1 and FFA4 are available, however, we should carefully validate these antibodies in terms of selectivity since the antibodies for GPCR sometimes lack its selectivity (34). Further, the specific agonist that shows the selectivity for specific signaling pathway such as biased ligand could be useful not only for the pharmacological tool but also for the therapeutic candidates. The adequate screening system for each signal pathway and structure–activity relationships of FFA1 and FFA4 ligand might be useful to develop these compounds. Further using this information, we could discriminate the beneficial effects of the FFA1 and FFA4 ligands from its adverse effects.

## Conclusion

**Figure 1:**
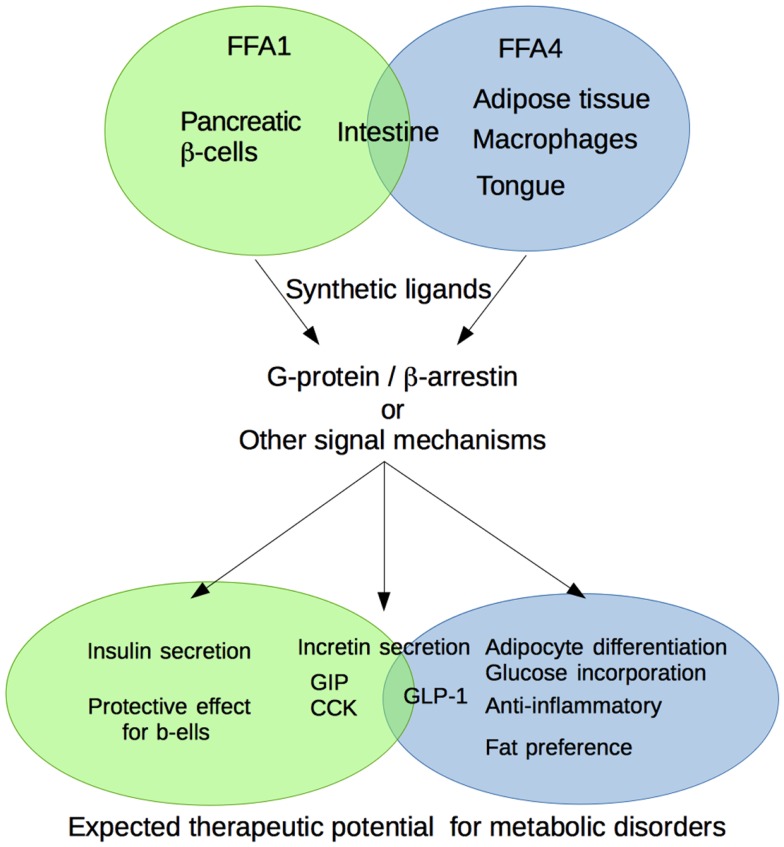
**Schematic diagram of the physiological function of medium- to long-chain fatty acid receptors FFA1 and FFA4 related to the metabolic regulation**.

FFA1 and FFA4 regulate the energy metabolic mechanism by acting as sensors for FFAs mainly provided by foods and lipolysis. A great number of reports showed that FFA1 and FFA4 are regulated by various physiological processes and the possibility for the therapeutic utility for metabolic disorders (Figure 1). However, we should reveal more precise molecular mechanisms underling metabolic diseases regulated by these receptors. In addition, the synthetic ligands, which can show selectivity not only among these receptors but also for the specific signals in each receptor, are expected to be developed in the future. Therefore, FFA1 and FFA4 may represent a promising therapeutic utility for the treatment of metabolic syndromes, such as obesity and diabetes.

## Conflict of Interest Statement

The authors declare that the research was conducted in the absence of any commercial or financial relationships that could be construed as a potential conflict of interest.
